# The Impact of Sex and Arterial Stiffness Interactions on the Outcome after an Acute Ischemic Stroke: A Retrospective Cohort Study

**DOI:** 10.3390/jcm13144095

**Published:** 2024-07-13

**Authors:** Maurizio Acampa, Pietro Enea Lazzerini, Alessandra Cartocci, Ernesto Iadanza, Gabriele Cevenini, Carlo Domenichelli, Riccardo Accioli, Viola Salvini, Francesca Guideri, Rossana Tassi, Giuseppe Martini

**Affiliations:** 1 Stroke Unit, Department of Medical Sciences, Surgery and Neurosciences, University of Siena, 53100 Siena, Italy; francesca.guideri@unisi.it (F.G.); giuseppe.martini@unisi.it (G.M.); 2 Electroimmunology Unit, Division of Internal Medicine and Geriatrics, Department of Medical Sciences, Surgery and Neurosciences, University of Siena, 53100 Siena, Italy; lazzerini7@unisi.it (P.E.L.); accioli.riccardo@gmail.com (R.A.); viola.salvini@unisi.it (V.S.); 3 Department of Medical Biotechnologies, University of Siena, 53100 Siena, Italy; alessandra.cartocci@gmail.com (A.C.); ernesto.iadanza@unisi.it (E.I.); gabriele.cevenini@unisi.it (G.C.); 4 Stroke Unit, Department of Emergency-Urgency and Transplants, Azienda Ospedaliera Universitaria Senese, “Santa Maria alle Scotte” General Hospital, 53100 Siena, Italy; carlo.domenichelli@ao-siena.toscana.it (C.D.); r.tassi@ao-siena.toscana.it (R.T.)

**Keywords:** ischemic stroke, arterial stiffness, outcome, cardiovascular risk factor, sex

## Abstract

**Background/Objectives**: Arterial stiffness (AS) is an independent predictor of cardiovascular events and is associated with a poor prognosis. While AS may represent a novel therapeutic target, recent evidence shows that it is sexually dimorphic. The aim of this study was to evaluate relative sex differences in arterial stiffness and their possible impact on the outcome of acute ischemic stroke. **Methods**: We retrospectively evaluated a cohort of adult patients with the following inclusion criteria: acute ischemic stroke, which occurred within 24 h from the onset of symptoms, confirmed through neuroimaging examinations, additional evaluations including extracranial and transcranial arterial ultrasound examinations, transthoracic echocardiography, a 12-lead resting ECG, and continuous 24 h in-hospital blood pressure monitoring. Based on the 24 h blood pressure monitoring, the following parameters were evaluated: systolic blood pressure, diastolic blood pressure, mean blood pressure, pulse pressure, and arterial stiffness index (ASI). The modified Rankin scale (mRS) was assessed at 90 days to evaluate the 3-month clinical outcome, defining an unfavorable outcome as an mRS score ≥ 3. To assess the factors associated with unfavorable outcomes, a stepwise logistic regression model was performed on the total sample size, and the analyses were replicated after stratifying by sex. **Results**: A total of 334 patients (176 males, 158 females) were included in the analysis. There was a significant sex-dependent impact of ASI on the 90-day unfavorable Rankin score (mRS score ≥ 3) as only men had a reduced likelihood of favorable outcomes with increasing arterial stiffness (OR:1.54, 95% CI: 1.06–2.23; P-interaction = 0.023). **Conclusions**: The influence of ASI on the 3-month functional outcome after acute ischemic stroke is at least in part sex-related, suggesting that, in males, higher ASI values are associated with a worse outcome.

## 1. Introduction

Arterial stiffness (AS) refers to the loss of arterial compliance associated with changes in vessel wall properties, resulting in a reduced ability of the arteries to expand and contract in response to pressure changes. Arterial stiffness results from a degenerative process affecting mainly the extracellular matrix (ECM) of elastic arteries [1] and independently predicts cardiovascular risk, representing a possible therapeutic target to improve the global burden of cardiovascular disease [2]. In particular, in patients affected by ischemic stroke, an increased AS represents a predictor of hemorrhagic transformation following thrombolysis or thrombectomy [3], and it is associated with a poor prognosis [4]. Thus, it has been proposed that AS may represent a novel therapeutic target for these patients [5].

Recent evidence showed that AS is sexually dimorphic [6]: young women have lower stiffness than age-matched men, but this sex difference reverses during normal aging. Despite these differences, AS seems to be an independent predictor of major adverse cardiovascular events also in women [7]; however, there are no studies about the relationships between sex and the influence of AS on functional outcomes after stroke.

In this view, the aim of our study was to evaluate possible relative sex differences in arterial stiffness and its possible impact on the outcome of acute ischemic stroke patients.

## 2. Materials and Methods

We retrospectively reviewed the medical records of adult patients admitted consecutively to our Stroke Unit (Siena University Hospital) for acute ischemic stroke (within 24 h from the onset of symptoms) from January 1, 2016, to January 1, 2019. The inclusion criteria were as follows: (1) acute ischemic stroke, occurring within 24 h from symptom onset; (2) confirmation by neuroimaging (brain CT with angio-CT scan and/or brain MR); and (3) comprehensive diagnostic work-up, comprising extracranial and transcranial arterial ultrasound examination, transthoracic echocardiography, 12-lead ECG, and continuous 24 h in-hospital blood pressure (BP) monitoring. Their neurological status at admission was assessed using the National Institute of Health Stroke Scale score (NIHSSs). Stroke pathogenesis was classified according to the TOAST criteria [8]. The modified Rankin scale (mRS) was assessed at 90 days by a stroke-trained physician, to evaluate the 3-month clinical outcome (defining an unfavorable outcome as an mRS score ≥ 3 to identify functional dependency) [9]. The study was approved by the Ethics Committee of the University Hospital of Siena, Italy.

### 2.1. Arterial Stiffness Index

According to our previous papers [10,11], BP monitoring was performed using validated oscillometric devices (Bedside Monitor Life Scope I BSM-2303K, International Div., Nihon Kohden Corp., Tokyo, Japan), in the first 24 h from admission to the hospital. BP was recorded for 24 h every 30 min, between 06:00 h and midnight, and hourly, from midnight to 06:00 h. For inclusion in the study, at least 80% of valid BP and heart rate measurements for each subject were needed. The following parameters were evaluated: systolic BP (SBP); diastolic BP (DBP); mean BP (MBP); pulse pressure (PP); and heart rate (HR). By plotting the individual values of SBP and DBP measurements, obtained through 24 h non-invasive monitoring, the linear regression slope of DBP on SBP was obtained and assumed as a global measure of arterial compliance, and its complement (1 minus the slope), named ASI, has been taken as a measure of arterial stiffness [12].

### 2.2. Statistical Analysis

All results are presented as means ± SD or as absolute frequencies and percentages. The normal distribution of quantitative variables was preliminary tested using the Kolmogorov–Smirnov test. Patients were dichotomized into two groups according to the sex. A Mann–Whitney test was performed to compare age, baseline NIHSS scores, and 3-month Rankin scores in both groups. A *t*-test was performed to compare hemodynamic parameters in both groups. The χ^2^ test was performed to evaluate the association between categorical variables (cardiovascular risk factors, favorable outcome, mortality) in both groups of patients. To assess the factors associated with unfavorable outcomes, we separately performed 2 different stepwise logistic regression analyses in women and men, adjusting for age, ASI, PP, MAP, NIHSS score, pathogenic subtype, AF, smoking, diabetes, hypertension, and the type of acute treatment. An additional third multivariate regression analysis was performed in the whole cohort, also including all the previous factors as independent variables. Furthermore, we included an interaction term (ASI × sex) in the logistic regression models and an interaction term between PP and MBP (PP × MBP) to consider possible collinearities. Area under the ROC curves (AUROC) were estimated to assess the predictability of ASI and of the model. Statistical analysis was performed using R Statistical Software (version 4.2.1 for Mac, R Foundation for Statistical Computing, Vienna, Austria).

## 3. Results

### 3.1. Baseline Characteristics

A total of 334 patients met the inclusion criteria and were included in the analysis (158 females [47%] and 176 men [53%]) (Supplemental Figure S1). Women were older (76 ± 13 vs. 72 ± 13 years, *p* = 0.009), had a higher prevalence of atrial fibrillation, and had a lower prevalence of smoking habit. In terms of the pathogenic subtype of ischemic stroke, in women, cardioembolic strokes were more frequent, and atherosclerotic strokes were less frequent (Table 1). The NIHSSs at the time of admission to the hospital was similar in both groups, and hemodynamic parameters showed no significant differences, including ASI. No statistical difference in antihypertensive treatment or in rehabilitation was observed when comparing men and women (Table 1).

### 3.2. Three-Month Functional Outcome and Mortality in the Whole Cohort

Women had a significantly higher percentage of unfavorable functional outcomes (mRS ≥ 3) in comparison with men, but the 3-month mortality was similar in both groups (Table 1). In the entire cohort, unfavorable outcomes were associated with several key factors, including advanced age, female gender, higher NIHSS scores upon admission, the presence of hypertension, diabetes, atrial fibrillation, cardioembolic pathogenesis, elevated SBP, DBP, MBP, and PP values, a greater frequency of diuretic use, reduced IV alteplase administration, and a higher incidence of rehabilitation therapy (Table 2).

When focusing specifically on women who experienced unfavorable outcomes (Table 3), distinct differences became apparent. This group tended to be older, presented with higher NIHSS scores upon admission, exhibited a higher prevalence of hypertension and diabetes, and were less likely to be smokers. Additionally, their hemodynamic parameters indicated higher SBP, DBP, MBP, and PP values compared to women with favorable outcomes. Moreover, women with unfavorable outcomes received intravenous alteplase treatment less frequently and necessitated rehabilitation therapy more often.

On the other hand, men who experienced unfavorable outcomes (Table 4) also displayed significant distinctions from those with favorable outcomes. These men were typically older, had higher NIHSS scores at admission, exhibited a higher prevalence of atrial fibrillation, and displayed higher SBP, PP, and ASI values in terms of hemodynamic parameters when compared to their counterparts with favorable outcomes. Like women, men with unfavorable outcomes were less likely to receive intravenous alteplase treatment and more likely to require rehabilitation therapy.

A multivariable logistic regression model was constructed to determine the factors associated with unfavorable outcomes. The best subset of associated variables for unfavorable outcomes were ASI (OR = 1.28 for an increase of 0.1, 95% CI: 1.01–1.61; *p* = 0.04), age (OR = 1.05 for an increase of 1 year, 95% CI: 1.02–1.09; *p* = 0.003), and NIHSSs (OR = 1.11 for an increase of 1 point, 95% CI: 1.07–1.17; *p* < 0.001) for men, and age (OR = 1.30 for an increase of 5 years, 95% CI: 1.16–1.47; *p* = 0.003), NIHSS score (OR = 1.12 for an increase of 1 point, 95% CI: 1.06–1.19; *p* < 0.001), diabetes (OR = 8.58, 95% CI: 2.74–33.15; *p* = 0.001), and MBP (OR = 1.06 for an increase of 1 mmHg, 95% CI: 1.02–1.11; *p* = 0.002) for women. To better investigate the role of ASI in both sexes, the ROC curves were performed. The AUROC of ASI is 0.63 (95% CI: 0.54–0.71) for male patients and 0.52 (95% CI: 0.43–0.61) for female patients.

### 3.3. Interaction between Sex and Arterial Stiffness

The correlation between all the covariates and the dependent variable was tested separately for men and women. All the covariates that resulted in differences between men and women were included as independent variables in the regression model. A stepwise multivariable logistic regression model was constructed to determine the factors associated with unfavorable outcomes in the whole cohort of patients, adjusting for sex and including an interaction term (ASI × male sex) in the logistic regression models. The best subset of associated variables for unfavorable outcomes were male sex, diabetes, age, MBP, NIHSS, and ASI × male sex (Table 5). There was a significant sex-dependent impact of ASI on 90-day unfavorable outcomes only in men (OR = 1.54 for an increase of 0.1, 95% CI: 1.06–2.23; P-interaction = 0.023). The AUROC of this model is 0.83 (95% CI: 0.79–0.87).

Given the heightened risk of atrial fibrillation development among individuals with arterial stiffness [13] and the compounded adverse prognosis when both conditions coexist [14], the same subset of variables was tested against a sub-dataset consisting only of those patients with sinus rhythm. All the variables found as significant from the whole model confirmed their significance, except for MBP, which stayed significant only for women. Arterial stiffness was specifically tested, finding no statistically significant difference between patients with atrial fibrillation and patients with sinus rhythm.

## 4. Discussion

The novel finding of our study is the following: there is a sex-dependent impact of ASI on functional outcomes as men have a higher likelihood of unfavorable outcomes with high ASI compared with women. Sex differences in ischemic stroke outcomes have been reported in many previous studies; it is clear that women have poorer outcomes than men, based on various different parameters including not only the severity of the neurological deficit at admission (based on the NIHSS score) but also other factors, such as age, pathogenic mechanisms (higher rates of cardioembolic strokes), pre-stroke functional status, and post-stroke depression [15]. In our cohort, even if there were no significant differences between males and females in terms of NIHSS score at admission and the pre-morbid mRS score (Table 1), it is noteworthy that cardioembolic strokes were more prevalent among women, who were also on average older than men. These distinctions may provide insights into the potentially worse functional outcomes observed in women.

Moreover, our findings hold particular significance as they underscore the varying influence of a specific cardiovascular risk factor, such as arterial stiffness, on post-stroke outcomes in both women and men. Previous studies demonstrated that various factors such as aging, obesity, hypertension, hormonal status, diet, and exercise can differently influence vascular stiffness in women and men [16]. It is unclear how the deleterious effects of AS may differ between females and males; however, previous studies compared the arterial ECM in both groups, showing that aging has a greater effect on ECM stiffness in men [6], and a previous study showed similar levels of arterial stiffness in aged male and female monkeys, but a decreased level of elastin only in males [17], suggesting that there may be unexplained differences in arterial structure or function between men and women despite similar levels of AS [18].

Recent evidence showed that increased stiffness is associated with poor performance on executive function tasks among males only [19], and executive function represents a strong predictor of recovery from disability in patients with acute stroke [20]. Moreover, negative associations between AS and composite global cognition were observed only in males [21].

Our study presents some limitations, including retrospective design and a lack of information on the impact of antihypertensive drugs that may affect ASI. An additional limitation of our study is that arterial stiffness (AS) was assessed using the arterial stiffness index (ASI). Although ASI offers the advantage of being easily measured with regular devices for 24 h blood pressure monitoring, it serves as a surrogate measure rather than a direct measure of AS. Currently, AS can be assessed through a variety of noninvasive procedures [22]. The gold standard for assessing AS is pulse wave velocity [23], and more recently, the cardio-ankle vascular index (CAVI) has also been actively studied, including in stroke patients [24]. Furthermore, we may not account for potential additional confounders such as hormonal status, dietary patterns, and exercise habits that can influence sex differences in the relationships between arterial stiffness and the associated cardiovascular risk.

Our results suggest a different impact of some cardiovascular risk factors, like arterial stiffness, in women and men with ischemic stroke; these differences could potentially indicate different therapeutic targets depending on sex; however, further studies are necessary in order to confirm and clarify the underlying pathophysiology of these differences.

## 5. Conclusions

The impact of ASI on 3-month functional outcomes after acute ischemic stroke is at least in part sex-related, suggesting that only in males, higher ASI values are associated with a worse outcome.

## Figures and Tables

**Table 1:** Sex-related clinical characteristics, cardiovascular risk factors, and hemodynamic parameters in patients with ischemic stroke.

	Women (*n* = 158)	Men (*n* = 176)	*p* Value
Age (years)	76 ± 13	72 ± 13	**0.009**
NIHSSs at admission	14 ± 8	13 ± 9	0.1
Cardiovascular risk factors			
Hypertension, *n* (%)	124 (78%)	122 (69%)	0.06
Diabetes mellitus, *n* (%)	35 (22%)	42 (24%)	0.7
Atrial fibrillation, *n* (%)	61 (39%)	49 (28%)	**0.04**
Hypercholesterolemia, *n* (%)	75 (47%)	74 (42%)	0.3
Previous CAD, *n* (%)	15 (9%)	26 (15%)	0.18
Previous stroke/TIA, *n* (%)	25 (16%)	40 (23%)	0.12
Smoking, *n* (%)	21 (13%)	70 (40%)	**<0.001**
TOAST classification			**0.002**
Atherosclerosis, *n* (%)	21 (13%)	51 (29%)	**0.005**
Cardioembolism, *n* (%)	75 (48%)	58 (33%)	**0.007**
Small vessel disease, *n* (%)	5 (3%)	11 (6%)	0.2
Other definite causes, *n* (%)	5 (3%)	7 (4%)	0.7
Cryptogenic strokes, *n* (%)	52 (33%)	49 (28%)	0.3
Hemodynamic parameters			
Systolic BP (mmHg)	137 ± 17	134 ± 17	0.1
Diastolic BP (mmHg)	70 ± 10	71 ± 10	0.6
Mean BP (mmHg)	94 ± 12	94 ± 12	0.9
Pulse pressure (mmHg)	67 ± 16	64 ± 15	0.08
ASI	0.64 ± 0.15	0.65 ± 0.13	0.6
Acute therapy			0.2
Intravenous thrombolysis, *n* (%)	78 (49%)	86 (49%)	
Mechanical thrombolysis, *n* (%)	42 (27%)	35 (20%)	
Combined therapy, *n* (%)	34 (21%)	46 (26%)	
Antihypertensive therapy	107 (68%)	102 (58%)	0.07
ACE-inhibitors/sartans, *n* (%)	71 (45%)	80 (45%)	1
Diuretics, *n* (%)	46 (29%)	45 (25%)	0.5
Beta-blockers, *n* (%)	47 (30%)	37 (21%)	0.08
Calcium-channel blockers, *n* (%)	18 (11%)	27 (15%)	0.3
Alpha-blockers, *n* (%)	3 (2%)	10 (6%)	0.09
Rehabilitation, *n* (%)	118 (76%)	137 (78%)	0.5
Premorbid Rankin score ≥ 3, *n* (%)	11 (7%)	8 (5%)	0.3
Outcome			
3-month Rankin score ≥ 3, *n* (%)	92 (58%)	79 (45%)	**0.01**
3-month mortality, *n* (%)	19 (12%)	15 (8%)	0.3

Data are expressed as mean ± SD or frequency counts and percentages. NIHSSs: National Institute of Health Stroke Scale score. CAD: coronary artery disease. TIA: transient ischemic attack. BP: blood pressure. ASI: arterial stiffness index. Bold values are statistically significant (*p* < 0.05).

**Table 2:** Clinical characteristics, cardiovascular risk factors, and hemodynamic parameters in patients with ischemic stroke, according to the functional outcome.

	mRS Score < 3 (*n* = 163)	mRS Score ≥ 3 (*n* = 171)	*p* Value
Age (years)	69 ± 14	78 ± 10	**<0.0001**
Sex (females/males)	66:97	92:79	**0.01**
NIHSS score at admission	10 ± 7	18 ± 8	**<0.0001**
Cardiovascular risk factors			
Hypertension, *n* (%)	111 (68%)	135 (79%)	**0.02**
Diabetes mellitus, *n* (%)	24 (15%)	53 (30%)	**0.0004**
Atrial fibrillation, *n* (%)	49 (30%)	83 (48%)	**0.0008**
Hypercholesterolemia, *n* (%)	74 (45%)	75 (44%)	0.8
Previous CAD, *n* (%)	16 (10%)	25 (15%)	0.1
Previous stroke/TIA, *n* (%)	28 (17%)	37 (22%)	0.3
Current smoker, *n* (%)	57 (35%)	34 (20%)	**0.002**
TOAST classification			**0.007**
Atherosclerosis, *n* (%)	36 (22%)	36 (21%)	0.8
Cardioembolism, *n* (%)	50 (30%)	83 (48%)	**0.001**
Small vessel disease, *n* (%)	11 (7%)	5 (3%)	0.1
Other definite causes, *n* (%)	8 (5%)	4 (3%)	0.2
Cryptogenic strokes, *n* (%)	58 (36%)	43 (25%)	**0.04**
Hemodynamic parameters			
SBP (mmHg)	131 ± 16	140 ± 17	**<0.0001**
DBP (mmHg)	69 ± 9	71 ± 11	**0.04**
MBP (mmHg)	92 ± 13	96 ± 11	**0.0006**
PP (mmHg)	61 ± 15	69 ± 16	**<0.0001**
ASI	0.63 ± 0.14	0.65 ± 0.14	0.09
Acute therapy			**<0.0001**
Intravenous thrombolysis, *n* (%)	102 (63%)	62 (36%)	**<0.0001**
Mechanical thrombolysis, *n* (%)	24 (15%)	53 (31%)	**0.0004**
Combined therapy, *n* (%)	34 (21%)	46 (27%)	0.2
Antihypertensive therapy	94 (57%)	115 (67%)	0.08
ACE-inhibitors/sartans, *n* (%)	73 (45%)	78 (45%)	0.9
Diuretics, *n* (%)	33 (20%)	58 (34%)	**0.006**
Beta-blockers, *n* (%)	36 (22%)	48 (28%)	0.2
Calcium-channel blockers, *n* (%)	20 (12%)	25 (15%)	0.6
Alpha-blockers, *n* (%)	6 (3%)	7 (4%)	1
Rehabilitation, *n* (%)	111 (68%)	144 (84%)	**0.0007**

Data are expressed as mean ± SD. NIHSS: National Institute of Health Stroke Scale. CAD: coronary artery disease. TIA: transient ischemic attack. SBP: systolic blood pressure. DBP: diastolic blood pressure. MBP: mean blood pressure. PP: pulse pressure. ASI: arterial stiffness index. Bold values are statistically significant (*p* < 0.05).

**Table 3:** Clinical characteristics, cardiovascular risk factors, and hemodynamic parameters in women with ischemic stroke, according to the functional outcome.

	mRS Score < 3 (*n* = 66)	mRS Score ≥ 3 (*n* = 92)	*p* Value
Age (years)	70 ± 14	80 ± 10	**<0.0001**
NIHSS score at admission	10 ± 6	17 ± 7	**<0.0001**
Cardiovascular risk factors			
Hypertension, *n* (%)	45 (68%)	79 (85%)	**0.01**
Diabetes mellitus, *n* (%)	5 (8%)	30 (33%)	**0.0002**
Atrial fibrillation, *n* (%)	25 (38%)	48 (52%)	0.1
Hypercholesterolemia, *n* (%)	28 (42%)	47 (51%)	0.3
Previous CAD, *n* (%)	3 (4%)	12 (13%)	0.09
Previous stroke/TIA, *n* (%)	9 (14%)	16 (17%)	0.6
Current smoker, *n* (%)	14 (21%)	7 (7%)	**0.01**
TOAST classification			0.2
Atherosclerosis, *n* (%)	8 (12%)	13 (14%)	
Cardioembolism, *n* (%)	25 (38%)	50 (54%)	
Small vessel disease, *n* (%)	3 (5%)	2 (2.5%)	
Other definite causes, *n* (%)	3 (5%)	2 (2.5%)	
Cryptogenic strokes, *n* (%)	27 (40%)	25 (27%)	
Hemodynamic parameters			
SBP (mmHg)	130 ± 17	141 ± 16	**<0.0001**
DBP (mmHg)	68 ± 10	71 ± 11	**0.04**
MBP (mmHg)	89 ± 11	97 ± 11	**<0.0001**
PP (mmHg)	62 ± 15	70 ± 17	**0.003**
ASI	0.65 ± 0.13	0.64 ± 0.16	0.4
Acute therapy			**0.01**
Intravenous thrombolysis, *n* (%)	40 (61%)	38 (41%)	**0.02**
Mechanical thrombolysis, *n* (%)	10 (15%)	32 (34%)	**0.006**
Combined therapy, *n* (%)	16 (24%)	18 (20%)	0.5
Antihypertensive therapy	39 (59%)	68 (74%)	0.06
ACE-inhibitors/sartans, *n* (%)	28 (42%)	43 (47%)	0.6
Diuretics, *n* (%)	15 (22%)	31 (33%)	0.1
Beta-blockers, *n* (%)	18 (27%)	29 (31%)	0.6
Calcium-channel blockers, *n* (%)	7 (10%)	11 (12%)	1
Alpha-blockers, *n* (%)	2 (3%)	1 (1%)	0.5
Rehabilitation, *n* (%)	42 (63%)	76 (82%)	**0.009**

Data are expressed as mean ± SD. NIHSS: National Institute of Health Stroke Scale. CAD: coronary artery disease. TIA: transient ischemic attack. SBP: systolic blood pressure. DBP: diastolic blood pressure. MBP: mean blood pressure. PP: pulse pressure. ASI: arterial stiffness index. Bold values are statistically significant (*p* < 0.05).

**Table 4:** Clinical characteristics, cardiovascular risk factors, and hemodynamic parameters in men with ischemic stroke, according to the functional outcome.

	mRS Score < 3 (*n* = 97)	mRS Score ≥ 3 (*n* = 79)	*p* Value
Age (years)	69 ± 13	77 ± 10	**<0.0001**
NIHSS score at admission	10 ± 7	18 ± 8	**<0.0001**
Cardiovascular risk factors			
Hypertension, *n* (%)	66 (68%)	56 (70%)	0.7
Diabetes mellitus, *n* (%)	19 (19%)	23 (29%)	0.1
Atrial fibrillation, *n* (%)	24 (25%)	35 (44%)	**0.009**
Hypercholesterolemia, *n* (%)	46 (47%)	28 (35%)	0.1
Previous CAD, *n* (%)	13 (13%)	13 (16%)	0.6
Previous stroke/TIA, *n* (%)	19 (20%)	21 (27%)	0.2
Current smoker, *n* (%)	43 (44%)	27 (34%)	0.2
TOAST classification			0.1
Atherosclerosis, *n* (%)	28 (29%)	23 (29%)	
Cardioembolism, *n* (%)	25 (26%)	33 (41%)	
Small vessel disease, *n* (%)	8 (8%)	3 (4%)	
Other definite causes, *n* (%)	5 (6%)	2 (3%)	
Cryptogenic strokes, *n* (%)	30 (31%)	18 (23%)	
Hemodynamic parameters			
SBP (mmHg)	131 ± 16	139 ± 17	**0.001**
DBP (mmHg)	70 ± 9	71 ± 11	0.4
MBP (mmHg)	94 ± 13	95 ± 9	0.6
PP (mmHg)	61 ± 15	67 ± 14	**0.003**
ASI	0.61 ± 0.14	0.67 ± 0.13	**0.002**
Acute therapy			**0.0001**
Intravenous thrombolysis, *n* (%)	62 (64%)	24 (30%)	**<0.001**
Mechanical thrombolysis, *n* (%)	14 (14%)	21 (27%)	0.06
Combined therapy, *n* (%)	18 (19%)	28 (35%)	**0.01**
Antihypertensive therapy	55 (57%)	47 (59%)	0.7
ACE-inhibitors/sartans, *n* (%)	45 (46%)	35 (44%)	0.8
Diuretics, *n* (%)	18 (18%)	27 (34%)	**0.02**
Beta-blockers, *n* (%)	18 (18%)	19 (24%)	0.4
Calcium-channel blockers, *n* (%)	13 (13%)	14 (17%)	0.5
Alpha-blockers, *n* (%)	4 (4%)	6 (7%)	0.3
Rehabilitation, *n* (%)	69 (71%)	68 (86%)	**0.01**

Data are expressed as mean ± SD. NIHSS: National Institute of Health Stroke Scale. CAD: coronary artery disease. TIA: transient ischemic attack. SBP: systolic blood pressure. DBP: diastolic blood pressure. MBP: mean blood pressure. PP: pulse pressure. ASI: arterial stiffness index. Bold values are statistically significant (*p* < 0.05).

**Table 5:** Factors associated with unfavorable outcomes (mRS ≥ 3) at 90 days in the whole cohort.

	OR	95% CI	*p* Value
Male sex	0.04	0.00–0.45	**0.01**
Diabetes mellitus	2.91	1.54–5.68	**0.001**
Age	1.30	1.16–1.47	**<0.0001**
MBP	1.03	1.00–1.05	**0.02**
NIHSSs at admission	1.13	1.09–1.17	**<0.0001**
ASI × male sex	1.54	1.06–2.23	**0.02**

NIHSS: National Institute of Health Stroke Scale score. MBP: mean blood pressure. ASI: arterial stiffness index. OR: odds ratio. CI: confidence interval. Bold values are statistically significant (*p* < 0.05).

## Data Availability

The data that support the findings of this study are available from the corresponding author upon reasonable request.

## Supplementary Materials

The following supporting information can be downloaded at: https://www.mdpi.com/article/10.3390/jcm13144095/s1, Figure S1: Flow chart.
