# Supplementary figures and images for: The Impact of Sex and Arterial Stiffness Interactions on the Outcome after an Acute Ischemic Stroke: A Retrospective Cohort Study
Source: J Clin Med. 2024 Jul 13;13(14):4095. doi: 10.3390/jcm13144095 (PMC11278401; doi:10.3390/jcm13144095)

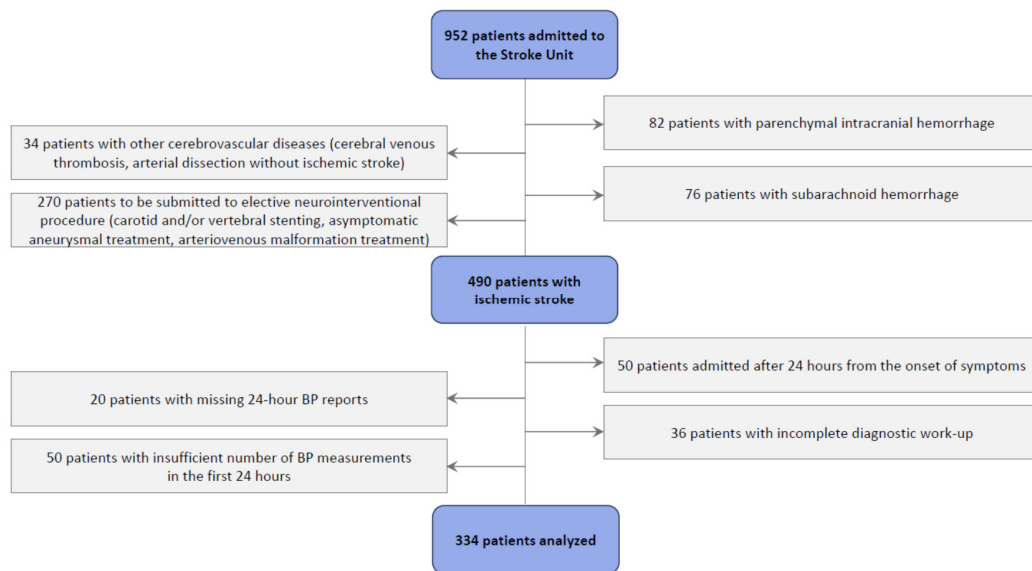

Figure S1: Flow chart.

Supplement: Supplementary file 1 [file jcm-13-04095-s001.zip › jcm-3060203-supplementary.pdf]
